# Low-intensity repetitive transcranial magnetic stimulation requires concurrent visual system activity to modulate visual evoked potentials in adult mice

**DOI:** 10.1038/s41598-018-23979-y

**Published:** 2018-04-11

**Authors:** Kalina Makowiecki, Andrew Garrett, Alan R. Harvey, Jennifer Rodger

**Affiliations:** 1 Experimental and Regenerative Neuroscience, The University of Western Australia, Crawley, Australia; 2 School of Biological Sciences, The University of Western Australia, Crawley, Australia; 3 School of Human Sciences, The University of Western Australia, Crawley, Australia; 4 Perron Institute for Neurological and Translational Science, Nedlands, Australia; 5 Present Address: Department of Systems Neuroscience, JFB, University of Goettingen, Göttingen, Germany

## Abstract

Repetitive transcranial stimulation (rTMS) is an increasingly popular method to non-invasively modulate cortical excitability in research and clinical settings. During rTMS, low-intensity magnetic fields reach areas perifocal to the target brain region, however, effects of these low-intensity (LI-) fields and how they interact with ongoing neural activity remains poorly defined. We evaluated whether coordinated neural activity during electromagnetic stimulation alters LI-rTMS effects on cortical excitability by comparing visually evoked potentials (VEP) and densities of parvalbumin-expressing (PV+) GABAergic interneurons in adult mouse visual cortex after LI-rTMS under different conditions: LI-rTMS applied during visually evoked (strong, coordinated) activity or in darkness (weak, spontaneous activity).We also compared response to LI-rTMS in wildtype and ephrin-A2A5^−/−^ mice, which have visuotopic anomalies thought to disrupt coherence of visually-evoked cortical activity. Demonstrating that LI-rTMS effects in V1 require concurrent sensory-evoked activity, LI-rTMS delivered during visually-evoked activity increased PV+ immunoreactivity in both genotypes; however, VEP peak amplitudes changed only in wildtypes, consistent with intracortical disinhibition. We show, for the first time, that neural activity and the degree of coordination in cortical population activity interact with LI-rTMS to alter excitability in a context-dependent manner.

## Introduction

Cortical processing of sensory-motor stimuli is important in many aspects of cognition and behaviour^1^. Cortical network excitability determines neuronal response and subsequent computations performed in processing sensory stimuli^2^. In primary sensory systems, intracortical inhibitory-excitatory circuits are critically important, because of their roles in processing stimulus characteristics and regulating activity-dependent plasticity of stimulus-specific responses^3^. In these recurrent intracortical circuits, inhibitory interneurons play a vital role by controlling individual pyramidal neuron response properties and therefore, regulating principal neuron output and signal propagation of sensory stimuli^4,5^. Additionally, intracortical circuits regulate activity across a distributed neural network to adjust population excitability and homeostatically stabilise network activity^6,7^. Regulation and modification of excitability is essential in learning and memory, and in development of normal sensory processing. However, uncontrolled excitability is linked to many neurological and psychiatric disorders^8–10^.

Non-invasive brain stimulation techniques, such as repetitive transcranial magnetic stimulation (rTMS), can induce persisting modifications of cortical excitability in humans, and have gained popularity as research tools, and in treatment of neurological and psychiatric disorders^11^. In rTMS, an electric current is induced in the brain via electromagnetic induction which, at sufficiently high intensities, evokes action-potentials in a proportion of neurons underlying the rTMS coil^12^. The stimulation-evoked spiking is thought to alter synaptic efficacy, resulting in excitability increases (long term potentiation, LTP-like) or decreases (long term depression, LTD-like), depending on the stimulation parameters (e.g. pulse frequency and intensity)^13,14^.

rTMS is often applied in humans at intensities termed “subthreshold”, referring to the use of motor threshold as a normaliser for variability between individuals^12,15^. Magnetic field intensities applied in rTMS are generally in the range of 0.3–2 Tesla (T)^13^. However, recent work suggests that lower field intensities in the range of 1–100 mT, which are far below motor threshold and do not directly evoke action-potentials, can affect neuron resting membrane potential^16^ and action-potential threshold^17^. Supporting this, low-intensity magnetic and electric fields improved depression symptoms in humans^18–20^. In animal models, low-intensity rTMS (LI-rTMS) induced beneficial structural plasticity, improving abnormal topography^21^, corrected deficits in visual-tracking behavior^22^, and increased intracellular Ca^2+^ in neurons *in vitro*^23–25^. Importantly, because low-intensity stimulation alters membrane electrophysiological properties and firing probabilities, without directly evoking action-potentials^17^, functional effects may be determined by interactions with evoked and/or stochastic neural activity during stimulation^26,27^. However, no previous studies have assessed immediate effects of LI-rTMS on excitability, or its interactions with network activity during stimulation.

In this study, we use the mouse visual system as a model to examine the contribution of neural activity during LI-rTMS to subsequent excitability changes. We recorded visual evoked potentials (VEPs), a reliable measure of cortical excitability^28,29^, from anesthetised mice, before and after 10 Hz LI-rTMS. We manipulated the balance between coordinated and stochastic cortical activity by: 1) the presence or absence of evoked responses, comparing effects of LI-rTMS during exposure to a visual stimulus, to LI-rTMS applied in the dark and 2) degree of coherence in the cortical response evoked by visual stimulation, by comparing wildtype to ephrin-A2A5 knockout (^−/−^) mice. Ephrin-A^−/−^ mice have grossly normal behaviour and visual-acuity^30,31^, but have topographic errors in primary visual circuits, with larger receptive fields and broader axon terminal zones, likely to contribute to lower overall coherence (e.g. decreased population of V1 neurons responding simultaneously) compared to wildtypes^32–34^. Finally, we tested whether LI-rTMS effects on excitability involve inhibitory system changes by analysing parvalbumin (PV+) cell densities in visual cortex. Parvalbumin is a calcium-buffering protein expressed in an inhibitory interneuron subpopulation which is particularly important in regulating cortical excitability. Furthermore, in rats, higher intensity rTMS modified PV expression^35–37^. We hypothesized that the type of activity during LI-rTMS would modulate its effects on intracortical inhibition-excitation balance, reflected in late VEP response components which are sensitive to cortical state and specifically, changes in GABAergic inhibition.

## Results

### VEP response characteristics

**Figure 1:**
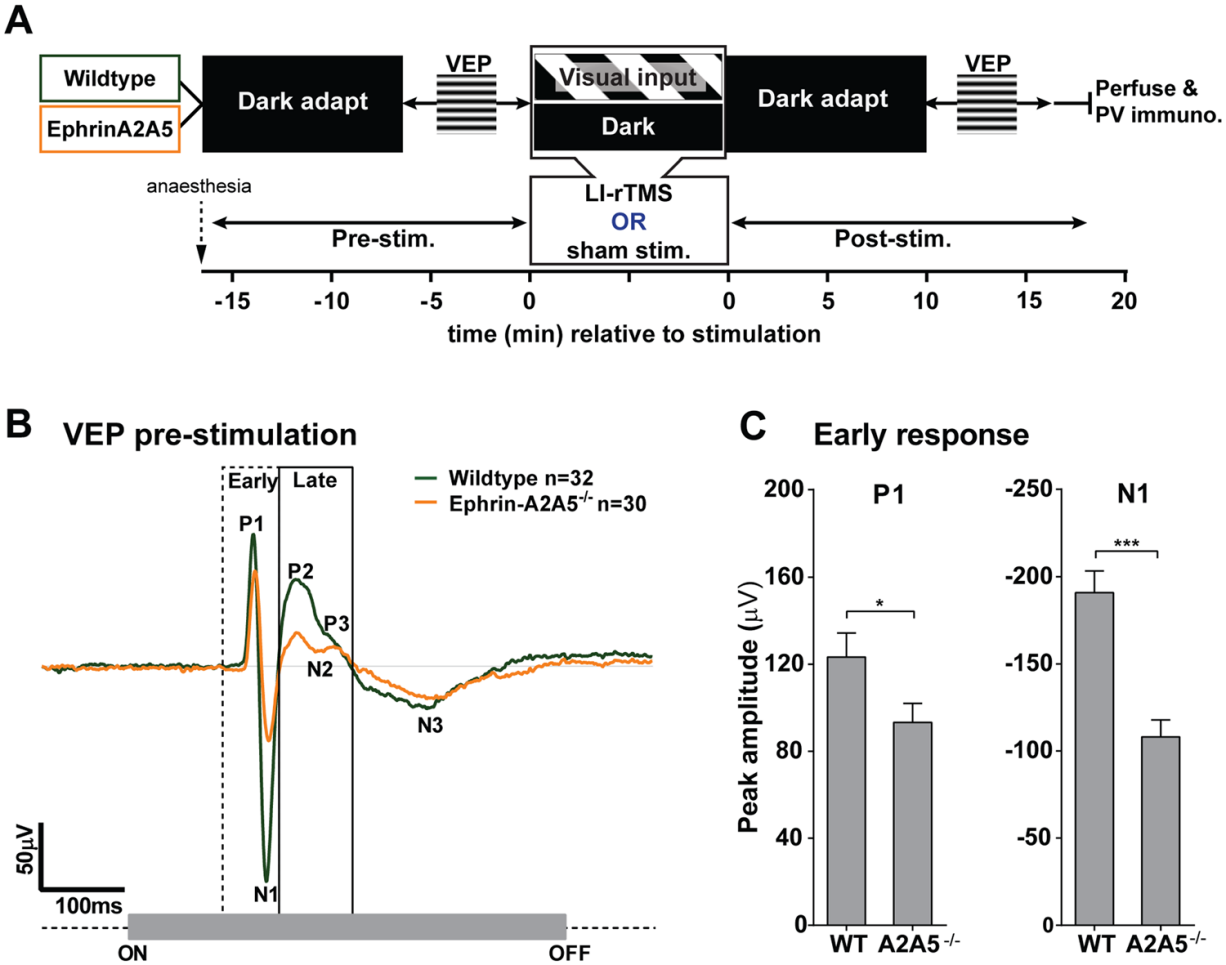
Ephrin-A2A5^−/−^ mice show smaller VEP peak amplitudes than wildtypes at pre-stimulation. (**A**) Schematic of experimental design. Mice were dark adapted for 10 minutes before recording pre-stimulation VEP responses to a flashed grating visual stimulus. Mice then received 10 minutes of 10 Hz LI-rTMS or sham (coil switched off control), either during exposure to a drifting grating visual stimulus (‘visual input’ condition) or in the dark. Mice then underwent a second 10 minute dark adaptation and post-stimulation VEP recording before being euthanised and processed for immunohistochemical staining for parvalbumin (PV). (**B**) Mean signal-averaged (32 flashes per trial, 4 trials) VEP traces for wildtype and ephrin-A2A5^−/−^ at pre-stimulation (delay groups only). Characteristic peaks are labelled, with demarcation for ‘early’ afferent VEP components (P1 and N1, ending at P2 onset) and ‘late’ (P2 onset to N3 onset) VEP response windows. The grey bar indicates the presentation of the visual stimulus. (**C**) Pre-stimulation mean (+SEM) peak amplitudes for early response peaks, P1 and N1. Asterisks indicate significance levels for Sidak corrected interaction analysis follow-up tests for simple effects of genotype at the pre-stimulation time point. Note reversed axis sign on N1 graph: up represents larger peak for both bar graphs. *p < 0.05, ***p < 0.001.

**Figure 2:**
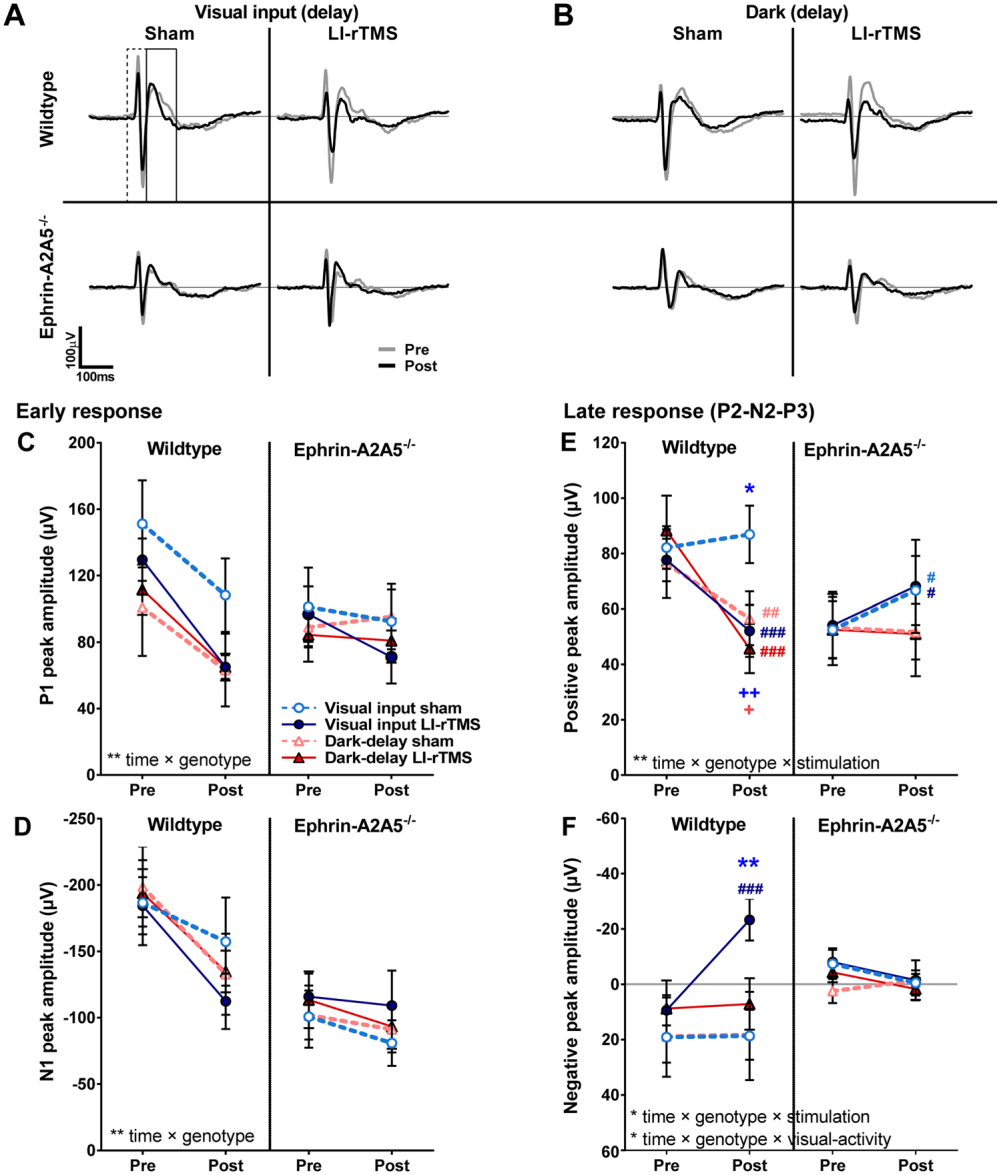
LI-rTMS affects late VEP response peak amplitudes only in wildtypes with concurrent visual input. (**A**) Mean VEP response to flashed grating after LI-rTMS or sham applied with visual input or (**B**) in the dark. Genotypes and stimulation conditions are shown separately with pre- and post-stimulation traces overlaid. Dashed line box represents boundaries defining ‘Early response’ window, and solid box represents ‘Late response’ window in which peak amplitudes were analysed (peak boundary windows were defined for each subject separately). (**C**–**F**) Pre- and post-stimulation mean (±SEM) peak amplitudes for (**C**) early response positive peak, P1, and (**D**) early response major negative peak, N1; (**E**) the largest positive peak in the late response window; and (**F**) largest negative peak within the late response window. Note reversed sign on negative peak axes: up represents larger peak on all graphs. Significant interactions (RM-ANOVA) are noted in each graph, with significance level denoted as: *p < 0.05, **p < 0.01. Coloured symbols show significance levels for each separate follow-up tests for simple effects (Sidak corrected). *p < 0.05, **p < 0.01, visual input groups sham vs. LI-rTMS; ^#^p < 0.05, ^##^p < 0.01, ^###^p < 0.001, pre-stimulation vs. post-stimulation, colour and position on graph denotes group; +p < 0.05, ++p < 0.01, change between pre- and post-stimulation LI-rTMS vs. sham (see also Fig. S1 for graphs expressing amplitudes as the difference between post- to pre-stimulation values).

We recorded visually evoked field potentials from anesthetised wildtype and ephrin-A2A5^−/−^ mice, before and after stimulation with LI-rTMS or sham (control, coil switched off), during visual input or in the dark (Fig. 1A). All mice showed characteristic surface-recorded VEP responses to the grating visual stimulus: a small positive peak (P1), a larger negative peak (N1), followed by more variable, alternating positive and negative deflecting peaks, designated as the P2-N2-P3 complex, and finally a ‘low and slow’ N3 peak (Fig. 1B). Positive waves recorded from the cortical surface generally correlate with a deep current ‘sink’ (excitatory postsynaptic potential, generating an inward current, resulting in negative extracellular potential) and simultaneous superficial ‘source’ (outward current at distance from the synapse, resulting in positive extracellular potential). P1 is generated from sources in the dLGN and sinks in layer 4 of the visual cortex, where geniculocortical afferents synapse. Surface negativity is correlated with a superficial sink and deep source^38^. N1 and N3 are generated from sinks in layer 4 with simultaneous layer 5 and 6 sources, indicating primary (N1) and rebound (N3) excitation of pyramidal cells by geniculocortical afferents^39^. Thus, P1, N1 and N3 are considered indicators of geniculocortical afferent input to the cortex. After P1, positivity corresponds to sources in layer 4 and sinks in layers 5 and 6, indicating inhibitory action on pyramidal cells. The alternating positive and negative peaks after N1, and well-defined P2-N2-P3 complex, are correlated with recurrent intracortical inhibition-excitation oscillations^38^. We found a difference in VEP peak amplitudes between genotypes; however, within each genotype, amplitudes at the pre-stimulation time point were similar between groups (p-values > 0.05, Fig. 2).

### Genotypes differ in VEP response pre-stimulation

Ephrin-A2A5^−/−^ mice at pre-stimulation had significantly smaller early response peaks (P1 and N1) compared to wildtypes (P1: p = 0.04, N1: p < 0.001, Fig. 1C). Smaller amplitudes indicate a less coherent population response due, for example, to fewer cells responding overall, or less synchronous response from the same size population. Ephrin-A2A5^−/−^ mice have ectopic retinogeniculate and geniculocortical projections that terminate outside topographically appropriate boundaries^33,34^. As such, lower afferent VEP response amplitudes are likely due to ectopic projections crossing representation boundaries of black-white cycles in the grating stimulus and firing asynchronously with neighbouring, topographically appropriate projections. This result supports the validity of ephrin-A2A5^−/−^ mice as a model to test how additional ‘noise’ with visually evoked activity during LI-rTMS affects excitability after stimulation. Therefore, keeping timing constant, we compared VEP peak amplitudes following LI-rTMS or sham applied during visual input, to VEP peak amplitudes following LI-rTMS or sham applied in the dark. Without visually evoked activity, geniculocortical and intracortical neurons fire spontaneously, but in adults spontaneous firing is not related to topographical accuracy of projections^40^, therefore, in darkness, activity is comparable between genotypes. Accordingly, we predicted that LI-rTMS in darkness would affect genotypes similarly, but when applied during visual input, we predicted it would interact with the different levels of activity coherence between genotypes.

### Early response components

Amplitudes of the early VEP components (P1, N1) were not significantly affected by stimulation or visual activity, either alone or in combination with genotype (RM-ANOVAs, interaction p-values > 0.05, see Table 1 for details). However, wildtype and ephrin-A2A5^−/−^ mice showed a significantly different pattern of change between pre- and post-stimulation (regardless of condition), with amplitudes decreasing significantly from pre-stimulation in wildtypes (follow-up analyses for wildtypes pooled across visual activity and stimulation groups, P1 and N1 p-values < 0.001, Fig. 2C,D), but not in ephrin-A2A5^−/−^ mice (p-values > 0.16).

**Table 1:** Interaction results from 4-way ANOVAs with factors: time (pre-, post-stimulation) and between-subject factors: visual activity, genotype and stimulation condition for VEP peak amplitudes.

		Early Response				Late Response			
		P1 (1,54)		N1 (1,54)		Positive peak (1,54)		Negative peak (1,45)	
		F	P	F	P	F	P	F	P
time ×	vis. activity × genotype × stim.	0.026	0.873	1.598	0.212	0.156	0.695	1.019	0.318
	genotype × stim.	0.010	0.922	0.497	0.484	**8.019**	**0.006**	**4.198**	**0.046**
	vis. activity × stim.	0.330	0.568	0.228	0.635	0.161	0.690	3.954	0.053
	vis. activity × genotype	0.172	0.680	0.116	0.735	0.299	0.587	**4.088**	**0.049**
	stim.	2.717	0.105	0.316	0.576	**8.081**	**0.006**	1.310	0.258
	genotype	**21.25**	**0.000**	**8.858**	**0.004**	**33.22**	**0.000**	**5.975**	**0.018**
	vis.activity	3.032	0.087	0.242	0.625	**15.05**	**0.000**	0.880	0.353

Statistically significant interactions shown in bold (to 3 decimal places). Degrees of freedom shown in parentheses.

### Visual activity during LI-rTMS affects late VEP response in wildtype but not ephrin-A2A5^−/−^ mice

To assess LI-rTMS effects on intracortical excitability, we analysed amplitudes of the largest positive peak, and largest (most negative) negatively-deflecting peak within the late VEP response window (see Methods), which correlate with intracortical inhibitory inputs and rebound pyramidal excitatory activity, respectively^38^.

Amplitudes of both the largest positive and the largest negatively-deflecting late response peaks were affected by LI-rTMS, but the response to LI-rTMS differed between wildtype and ephrin-A2A5^−/−^ mice and depended on visual activity during stimulation (Fig. 2E,F, RM-ANOVAs, significant 3-way and 2-way interactions, see Table 1 for statistics). Follow-up tests showed that only wildtypes with visual input were affected by LI-rTMS, with significantly reduced late response positive peak amplitudes compared to sham (p = 0.04; all other groups LI-rTMS vs. sham, p-values > 0.51) and compared to pre-LI-rTMS (p < 0.001). In contrast, wildtypes with sham stimulation (visual input alone) did not significantly change from pre-stimulation (p = 0.47). Keeping timing constant, wildtypes in the dark, regardless of sham or LI-rTMS showed significantly decreased amplitude of the late response positive peak compared to pre-stimulation (LI-rTMS: p < 0.001; sham: p = 0.004). The magnitude of decrease from pre-stimulation was significantly greater with LI-rTMS than sham (p = 0.02), but at post-stimulation LI-rTMS and sham groups were not significantly different (p = 0.51, Fig. 2E. See also Fig. S1, showing group means of within-subject change in amplitude from pre- to post-stimulation stimulation values).

Similarly, LI-rTMS effects on negatively-deflecting peak amplitudes were present only in wildtypes with visual input: LI-rTMS significantly increased the negatively-deflecting peak (amplitude decrease, more negative) compared to sham (p = 0.001) and compared to pre-LI-rTMS (p < 0.001, Fig. 2F). There were no significant changes in negatively-deflecting peak amplitudes from pre-LI-rTMS in dark-stimulated wildtypes, nor in ephrin-A2A5^−/−^ mice in any condition (p-values > 0.05). Furthermore, in wildtypes with LI-rTMS during visual input, the change in amplitude values from pre-stimulation to post-stimulation were strongly and significantly correlated between the positive and negative peak (r = 0.83, p = 0.02), indicating that decreases in the inhibitory positive peak between pre- and post-stimulation were associated with increases in the excitatory negative peak. Positive and negative peak amplitude changes from pre-stimulation were not significantly correlated in any other group (p-values > 0.05).

In contrast to wildtypes, LI-rTMS in ephrin-A2A5^−/−^ mice had no significant effect on either the positive or negative late response peak, regardless of visual activity during stimulation (p-values > 0.78). However, positive peak amplitudes significantly increased after visual input alone in ephrin-A2A5^−/−^ mice (p-values = 0.04), whereas the dark-stimulated groups showed no significant change relative to pre-stimulation (p-values > 0.82).

The mean VEP response traces show that the overall structure of the VEP waveform remained constant between pre- and post-stimulation (Fig. 2A,B). Peak latencies did not change significantly from pre-stimulation for any peak, and there were no significant interactions with the change from pre-stimulation by genotype, visual activity or stimulation (all p-values > 0.05). Furthermore, across groups, changes in late response peak amplitudes were not accompanied by altered number of peaks (Poisson regression: model effects of visual activity, genotype, stimulation and interactions, all p-values > 0.05). Likelihood of having at least one negatively-deflecting peak was not significantly different between groups at either pre- or post-stimulation (Pearson χ^2^ (7) < 6.55, p-values > 0.52). These results indicate that LI-rTMS effects on amplitude were not attributable to changes in late response peak definition (e.g. emergence/disappearance of the P2-N2-P3 complex), but instead occurred within consistently present peaks.

### Time in the dark and delay after stimulation

A caveat in comparing effects of LI-rTMS during visual input, to time-matched dark-stimulated animals, is that the visual input and dark-stimulated groups also differed in total time in the dark: the visual input cohort received 10 min LI-rTMS during visual input, followed by a further 10 min in the dark; the dark-stimulated cohort had identical timing: receiving 10 min LI-rTMS while in the dark, followed by a further 10 min still in the dark after stimulation ceased, thus the total time in the dark was 20 min. Because of this, the results presented above comparing the visual input and the dark-stimulated groups cannot *a priori* exclude an interaction between LI-rTMS and total time in darkness. Therefore, we tested another cohort of mice which received LI-rTMS or sham in the dark, and instead of matching the 10 min post-stimulation delay, VEPs from these mice were recorded *immediately* after LI-rTMS (dark-immediate cohort), thus keeping time in the dark consistent with the visual input cohort (see Methods and Supplementary Fig. 1A for design details). Note, it was not possible to perform an immediate group with visual input because VEP recordings require a period in the dark for adaptation^29^, see Methods.

Following the same analysis procedure as above for the dark-immediate cohort, VEP peak amplitudes did not change significantly from pre-stimulation for any group, and there were no significant interactions, indicating that LI-rTMS applied in the dark has no immediate effect on VEP peak amplitudes for either genotype (ANOVAs, all p-values > 0.05). We confirmed that this was not due to pre-existing differences between cohorts, because within each genotype, pre-stimulation measures were not significantly different between the dark-immediate, dark-delay and visual input-delay cohorts (p-values > 0.05). Therefore, the effects of LI-rTMS during visual input on late response peaks seen in wildtypes cannot be entirely due to the shorter period in the dark (10 mins) because the same pattern of results would have been seen in the dark-immediate group (also 10 mins in darkness). This result supports our hypothesis that visual input during stimulation alters LI-rTMS effects on VEP responses. Further analysis of the independent contributions of time in the dark and of delay after stimulation to the effects of LI-rTMS, visual activity and genotype are provided in Supplementary Information.

### Parvalbumin expression changes with LI-rTMS

**Figure 3:**
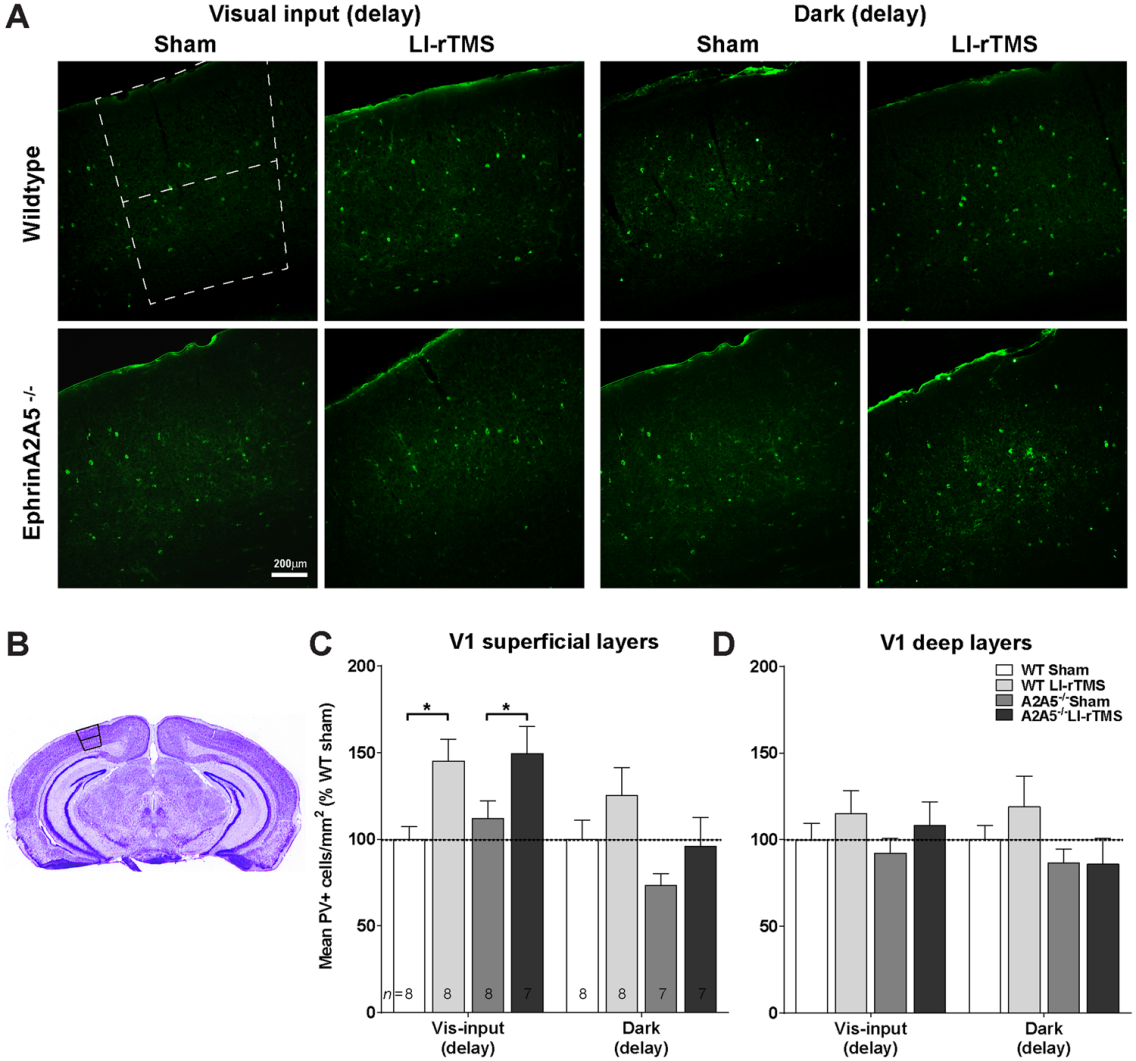
LI-rTMS effects on V1 parvalbumin immunoreactivity require visual input during stimulation. (**A**) Example images of V1 sections immunohistochemically labelled for parvalbumin-expressing (PV+) cells from animals of each genotype (wildtype, ephrin-A2A5^−/−^) and each stimulation condition (LI-rTMS or sham, applied during visual input from a drifting grating stimulus, or in the dark). Non-linear ‘curves’ adjustments were applied to reduce background and increase cell visibility in small figure panels (for figure presentation only; counts were performed without these adjustments). Scale bar applies to all images. (**B**) Nissl stained section showing V1 boundaries and the division between superficial (pia to layer 4) and deep (layers 5 and 6) cortical layers. V1 and cortical layers were identified on adjacent nissl stained sections to define regions for PV+ cell counts for both left and right sides and at least 3 sections per subject. Number of PV+ cells were divided by the area counted from for each section to obtain densities. (**C**) Mean (+SEM) PV+ cells densities, expressed as a percentage of wildtype sham within each visual activity condition, for superficial visual cortical layers and (**D**) deep visual cortical layers. *p < 0.05.

The parvalbumin-expressing subtype of fast-spiking GABAergic interneurons are particularly important in regulating cortical excitability via recurrent intracortical inhibition, associated with the late VEP components^41,42^. Therefore, we next assessed whether the different electrophysiological effects of LI-rTMS depending on visual activity and genotype involve intracortical inhibition, by comparing parvalbumin positive (PV+) cell densities in superficial and deep layers of visual cortex (Fig. 3). We assessed PV+ cell densities only in the delay cohorts because exposure to the visual stimulus used to elicit VEP responses for the post-stimulation recording meant that the dark-immediate cohort was not comparable to delay groups on either time in the dark or time since stimulation. We confirmed that PV+ densities did not significantly differ between visual activity types for wildtype-sham groups (ANOVA, p-values > 0.05), and therefore, PV+ density values were expressed as percentage of wildtype-sham within each visual activity group (visual input, dark) for subsequent analyses.

Stimulation condition and visual activity significantly affected PV+ cell densities. Within-subjects, effects of stimulation and visual activity also differed between superficial and deep cortical layers (ANOVA, layer × stimulation, F (1,53) = 14.17, p < 0.001, layer × visual activity, F = 16.50, p < 0.001). Contrasting with the VEP results, PV+ densities were not significantly different between wildtype and ephrin-A2A5^−/−^ mice, and there were no genotype-specific effects of LI-rTMS and visual activity (genotype interaction p-values > 0.05). Follow-up tests showed that LI-rTMS and visual activity significantly affected PV+ cell densities only in superficial cortical layers, with LI-rTMS during visual input resulting in significantly higher PV+ densities compared to sham in both wildtype (p = 0.01) and ephrin-A2A5^−/−^ mice (p = 0.04, Fig. 3C). Differently, LI-rTMS in the dark did not significantly affect PV+ densities in either genotype (LI-rTMS vs. sham, p-values > 0.15). PV+ cell densities were not significantly different between groups in deep cortical layers (p-values > 0.05, Fig. 3D).

We also analysed correlations between PV+ densities and magnitude change in VEP peaks between pre- and post-stimulation in individual animals. Although correlations for some groups were of moderate strength, they were non-significant (Supplementary Table 2).

## Discussion

We show LI-rTMS alters visual cortical excitability and demonstrate a key role of activity during stimulation in determining these effects. Differential effects of LI-rTMS applied with or without concurrent visual stimulus-evoked activity suggest that LI-rTMS may interact with summed population activity. Furthermore, LI-rTMS effects on visual system electrophysiological function may be sensitive to the degree of coherence in activity during stimulation (e.g. the proportion of neurons responding simultaneously): LI-rTMS during cortical activity with less coherence (in the dark, or in ephrin-A2A5^−/−^ mice) had no additional effects on intracortical inhibition-excitation balance. Interestingly, comparisons between wildtype and ephrin-A2A5^−/−^ mice revealed that electrophysiological effects of LI-rTMS on VEP responses were dissociable from its effects on parvalbumin immunoreactivity. Taken together, these results suggest that both activity and the precision of the underlying cortical circuitry contribute to LI-rTMS outcomes. The interactions between LI-rTMS and activity may then permit or facilitate longer-term structural reorganisation in neural projections and visual function improvements previously observed following LI-rTMS in ephrin-A2A5^−/−^ mice^21,22^.

### LI-rTMS affects late VEP response peaks

In contrast to the early VEP, the late VEP components were clearly modulated by LI-rTMS, with wildtypes showing decreased positive and increased negative peaks after LI-rTMS during visual input. Given that magnetic field intensity decreases with distance from the coil, the cortex receives relatively stronger stimulation than the thalamus. Thus, late VEP components reflecting intracortical activity (P2-N2-P3) may be more susceptible to LI-rTMS effects than the early VEP response components, which reflect geniculocortical afferent presynaptic input (P1) and postsynaptic excitatory response in layer 4 pyramidal cells (N1)^38,39^. The late response, and particularly the P2-N2-P3 complex, is largely driven by intracortical GABAergic inhibitory and rebound pyramidal excitatory oscillations, which become progressively more out of phase towards N3 (rebound pyramidal excitation)^38^. In wildtypes with visual input during LI-rTMS, the decrease in positive peak maximum amplitude in this window was accompanied by increased excitatory peak amplitude, consistent with disinhibition of GABAergic circuits, as has been reported for rat somatosensory cortex after rTMS^43^.

Our results are similar to those previously described for anodal tDCS aftereffects in humans, showing late VEP response amplitude decreased in the analogous late positive peak, and increased in the negatively-deflecting peak^44^. Furthermore, whisker-deflection evoked potentials in rat somatosensory cortex also showed greater changes in amplitudes of response peaks corresponding to intracortical, rather than thalamocortical components after rTMS with theta burst stimulation (TBS)^43^. We note that the stimulation intensity used by Thimm and Funke (2015) activated callosal axons to antidromically stimulate layer 2/3 pyramidal cells, whereas our stimulation does not elicit action potentials^17^. While the influence of stimulation parameters and differences between the visual and somatosensory systems cannot be excluded, considered together, these studies suggest that intracortical circuits may be particularly susceptible to modulation by electromagnetic stimulation, for example, due to spatial relationship of neurons relative to the magnetic field^45^ and/or lower activation threshold of inhibitory interneurons^46^. However, further experiments are needed to determine whether the altered balance of inhibitory-excitatory VEP peaks reported here indeed arise from disinhibition or increased excitatory output or a combination.

### LI-rTMS immediate effects on V1 excitability require evoked activity during stimulation

We show that LI-rTMS interacts with co-occurring brain activity during stimulation, because changes in intracortical VEP components were observed only in wildtypes with visual input: the condition with the strongest and most coordinated cortical activity during stimulation. Wildtypes receiving visual input during LI-rTMS had decreased amplitude of the positive peak within the late VEP response, and also showed a correspondingly larger negative peak. The negative peak correlates to excitatory pyramidal cell rebound activity, and an increase reflects either enhanced synchrony and/or number of cells responding simultaneously^38^. Taken together, these results suggest that LI-rTMS-induced decreases in positive peak amplitudes are unlikely to be an artefact of additional, random firing, but instead are consistent with increased excitation, possibly due to suppression of recurrent cortical inhibition as suggested for rTMS at 10Hz^47^. By contrast, a period in the dark, regardless of stimulation, decreased positive peak amplitude in wildtypes, without an accompanying increase in the excitatory peak, suggesting increased ‘noise’ in the response rather than disinhibition. Because wildtypes (sham) in the visual input groups did not change over time, this effect cannot be attributed to anaesthetic depth, habituation or other time-related factors, and could be due to time in the dark itself.

Increased stochastic firing from 20 minutes in the dark presents a possible explanation for absence of LI-rTMS effects on VEP and PV+ densities in dark-stimulated mice: further stochastic activity (noise), in the absence of synchronous evoked responses (signal), is unlikely to have an additive effect, over a brief period in the dark, on VEP response coherence or visual cortical excitability. Thus, this supports our model that LI-rTMS only increases probability of firing and therefore, without concurrent evoked activity, LI-rTMS only enhances stochastic activity^48^.

Our genotype-specific results further support that cortical activity plays a determinant role in LI-rTMS effects on cortical excitability, as recently proposed for human rTMS^49^. We found that LI-rTMS had no effect on VEP amplitudes in ephrin-A2A5^−/−^ mice in which the cortical response to evoked activity is less coherent than in wildtypes^32,33^. Furthermore, when spontaneous firing was likely equivalent between genotypes, the genotype-specific effects of LI-rTMS were absent. These findings suggest that coherence of brain activity may play a role in differential effects of LI-rTMS between normal and abnormal systems previously shown in mice^21,22,50^ and in healthy compared to clinical populations in humans receiving higher intensity rTMS^51^. Moreover, this supports that baseline asynchrony in cortical oscillations may be an important determinant of clinical outcomes of non-invasive brain stimulation^49^.

### Dissociation between LI-rTMS effects on VEP and PV measures

Changes in the PV+ cell density were observed in both genotypes following LI-rTMS during visual input, and not in the dark, suggesting that the summed neural network activity level may be particularly important in LI-rTMS effects on the PV+ populations. A principal role of PV+ neurons is to prevent hyper-excitability by inhibiting pyramidal cell activity across a widely distributed network^4^. Though LI-rTMS induces only subtle changes to neuron membrane properties^17^, the small increase in firing probability induced by LI-rTMS together with visually evoked activity, may result in increased overall neuronal activity. Such an increase in network activity would feedback to increase PV+ interneuron inhibitory activity, and therefore downregulate population activity^2^. In the dark the overall network activity is lower^52^, therefore an increase in firing probability by LI-rTMS alone may not be sufficient to trigger PV+-mediated inhibition.

### Activity-dependent changes to inhibition may reflect calcium-buffering gating mechanisms in LI-rTMS effects

We observed increases in PV+ labelling in mice perfused approximately 20 minutes after the end of stimulation, which is in line with effects of higher intensity rTMS in rats, showing an initial increase in cortical PV+ cells 6.5 min after ending stimulation, however, PV+ cell numbers decreased at 35 minutes^53^. Further investigations are needed to assess whether PV+ density changes after LI-rTMS follow a similar non-linear u-curve. However, the short time course of this experiment excludes PV synthesis as an explanation for increased PV+ cell densities^54,55^. It is possible that increased PV+ cell density reflects reduced degradation, however a more likely explanation is that immunoreactivity has increased due to an increased amount of Ca^2+^ bound to the protein^56^. We previously showed LI-rTMS increased Ca^2+^ concentrations in cultured (predominantly inhibitory) cortical neurons from intracellular sources^25^. Considered together with results of the current study, this suggests that the increased PV+ density following LI-rTMS *in vivo* reflects Ca^2+^ increases caused by the combination of stimulation and visual system activation.

We previously showed that LI-rTMS delivered daily over 14 days induced structural plasticity in adult ephrin-A2A5^−/−^ mice, improving visual system topographical organisation across multiple synaptic relays^21,22^. This long term (days) plasticity likely involves multiple mechanisms over different time scales, including changes in gene expression and neuromodulators^14,25^, postsynaptic receptor expression^57^, and extracellular proteoglycan matrix composition, which normally limits adult plasticity^58,59^. Results of the current study now add to understandings of the short-term effects of LI-rTMS, demonstrating electrophysiological and PV+ immunoreactivity effects consistent with altered presynaptic GABAergic release, mediated by PV-calcium buffering. PV-calcium buffering controls timing of neurotransmitter release in inhibitory synapses to pyramidal cells, which is integral in regulating short-term and spike timing-dependent plasticity^60–62^. Considered together with previous *in vitro* evidence showing that LI-rMS increased pyramidal spiking^17^, the changes we found in wildtype VEP peaks indicating intracortical disinhibition suggest that LI-rTMS may increase ‘noise’ during visual input – i.e. signals that are temporally uncorrelated with neighbouring neurons. An increase in spontaneous spiking across the neural population increases opportunity for spike timing dependent-plasticity, via enhancement of Hebbian synaptic competition and detection of asynchrony, triggering selective weakening of aberrant projections^63^. Though future studies are needed to directly measure LI-rTMS effects on calcium buffering and inhibition, these initial results suggest a possible presynaptic mechanism underlying, or contributing to, the selectivity for abnormal projections previously found in ephrin-A2A5^−/−^ mice after chronic stimulation^21,22^.

## Conclusions

We demonstrate, for the first time, that LI-rTMS can induce changes in visual cortical excitability and PV immunoreactivity, but these effects require evoked activity during stimulation. Moreover, comparisons between wildtype and ephrin-A2A5^−/−^ mice revealed a dissociation between LI-rTMS effects on electrophysiological and inhibitory interneuron calcium-buffering protein expression, suggesting that coherence of neural activity is important in excitability following stimulation, but increases in PV immunoreactivity by LI-rTMS may be independent of VEP responses. The implication is that LI-rTMS may have persisting impact on the molecular profile of a population of inhibitory interneurons, but electrophysiological effects of LI-rTMS, in the timeframe investigated, may require concurrent activity that is both strong and highly coordinated, as occurs for visually evoked activity in wildtypes.

Though there are obvious limitations to the extent that results in a mouse model can be generalised to humans, this study highlights that low-intensity fields, applied to brain regions deeper and peripheral to the target brain region in rTMS, may interact with rTMS-evoked spiking and contribute to its effects in clinical and healthy populations. This possible contribution could therefore be exploited in future therapeutic approaches to help tailor treatments to individuals.

## Methods

### Animals and Housing

This study used 48 wildtypes (C57/Bl6J, 26 males) and 47 ephrin-A2A5^−/−^ knockout mice (25 males). Wildtype mice were obtained from the Animal Resources Centre (Canning Vale, Australia). Ephrin-A2A5^−/−^ mice were originally a generous gift from David Feldheim and backcrossed for >10 generations on the same genetic background as wildtypes, and subsequently bred from heterozygous parents at Biomedical Research Facility at The University of Western Australia. Mice were genotyped at weaning, as described previously^64^. Mice were housed with a maximum of five mice per cage in standard cages (base 17 × 19 cm, height 16 cm, clear plastic) in a controlled environment (12/12 light/dark cycle, 22 °C ± 2°) with food and water *ad libitum*. All mice were at least 8 weeks old at the time of electrophysiological recordings, ensuring subjects were outside visual system developmental critical periods (~P35)^40,65^. Preliminary analyses confirmed no significant effects of age or sex on any measure (ANOVAs, all p-values > 0.05). All procedures in this study were approved by The University of Western Australia Animal Ethics Committee (approval number RA/100/1214) and conducted in accordance with NIH guidelines.

### Experimental design

LI-rTMS effects were tested in presence and absence of visual stimuli, in two genotypes (wildtype and ephrin-A2A5^−/−^). Each visual activity type/genotype condition included a sham (no stimulation) control group (Fig. 1A).

The experimental protocol was as follows: approximately 24 hours after surgery to implant electrodes, mice were anesthetised (see below), and dark adapted for 10 minutes prior to commencing VEP recordings (light in the room switched off). This duration is sufficient to avoid retinal bleaching effects^66^, and unlikely to induce excitability changes associated with longer periods of sensory deprivation^67^. ‘Pre-stimulation’ baseline VEP recordings were then performed, immediately followed by 10 minutes of either LI-rTMS or sham stimulation. LI-rTMS or sham stimulation was applied either during exposure to visual stimuli (‘visual input’ condition), or in the dark. Visual stimuli consisted of square wave drifting gratings (0.08 cycles per degree, 0.5 Hz drift velocity,100% contrast) at eight different orientation and direction combinations (0°, 90°, 45°, 135°) presented in random order. For comparability with VEPs at the pre-stimulation time-point, and to control for effects of a visual stimulus immediately before the VEP recordings (e.g. retinal bleaching), mice underwent a second 10 minute dark-adaptation period before the second (‘post-stimulation’) VEP recording. For mice receiving LI-rTMS/sham in the dark, timing was kept consistent with the visual input cohort, and mice underwent a further 10 minutes in the dark, for a total of 20 minutes without visual stimulation (‘dark-delay’ condition). To test whether time in the dark had an effect, we also included a cohort which underwent post-stimulation VEP recordings *immediately* after receiving LI-rTMS in the dark (‘dark-immediate’ condition). We did not include an equivalent group recorded immediately after visual input because exposure to visual stimuli without a period of dark adaptation affects the VEP response^29^, precluding comparison to the pre-stimulation time point within the same subjects and to other groups. Data from the dark-immediate group are shown in Supplementary Information.

### Electrodes

Recordings of visual evoked potentials were obtained from anesthetised mice using skull-implanted screw electrodes. Implantation of screw electrodes was as described previously^28^. Briefly, mice were anesthetised with intraperitoneal injection of ketamine (75 mg/kg, ketamine hydrochloride, Ilium; Troy Laboratories Pty. Ltd, Glendenning, NSW, Australia) and medetomidine (1 mg/kg, medetomidine hydrochloride, Ilium; Troy Laboratories Pty. Ltd)^68^, upon absence of foot-pinch withdrawal reflex, the head was shaved and the mouse secured in a stereotaxic frame and the dorsal surface of the skull exposed. Electrode positions were marked with ink on the skull surface overlying the right monocular primary visual cortex (3.6 mm caudal, 2.3 mm lateral from Bregma) and frontal cortex for the reference electrode (2 mm rostral, 0.5 mm lateral from Bregma)^69^. Pilot holes were hand-drilled with 0.5 mm diameter drill-bit (Model No: A3162 #114,731, Titex Drills; K2 Engineering, Perth, Australia). The stainless steel screws (M0.6 × 2 mm length; Micro Fastenings Ltd., West Sussex, UK) were inserted into pilot holes to contact the dura without penetrating into the cortex and secured in place with dental cement (Poly-F Plus; Densply GmbH, Mannheim, Germany) leaving the head of the screw exposed. Mice received the reversal-agent for medetomidine (atipamezole hydrochloride, 1 mg/kg, subcutaneous injection; Ilium, Troy Laboratories) and Xylocaine gel (2% lignocaine hydrochloride; AstraZeneca, North Ryde, Australia) applied to the scalp for topical analgesia. Mice were returned to their cages for ~24 hours to allow the dental cement to set completely. We previously established that the 24 hr recovery after electrode implantation and the anaesthetic protocol used here yields highly stable VEP responses over a ~40 minute recording period in wildtype mice without other interventions^28,70^. To use the implanted screws as electrodes, an artery clamp (Part No:18,052–03; Fine Science Tools, Foster City, CA) soldered to a chlorided silver wire (Part No:781,500; A-M Systems, Australia) was clipped to each screw during recordings. A chlorided silver grounding electrode was inserted into the skin near the tail with a 27 gauge needle.

### Visual evoked potential recordings

During recordings, mice were placed in a light-protected Faraday cage, and kept at constant body temperature using thermo-coupled heatpad (TC-1000; CWE Inc., Ardmore, PA). AC-coupled differential recordings were obtained using a DAM50 amplifier (World Precision Instruments, Sarasota, FL) via PowerLab and Scope software (v4.1.1, ADInstruments, Bella Vista, Australia). Data were sampled at 2 kHz, amplified (ac x10,000), bandpass and notch filtered (10–500 Hz and 50 Hz, respectively). The visual stimulus for VEPs was a sinusoidal grating (0° orientation, 0.16 cycles per degree, 50% contrast), generated using custom MATLAB scripts (Mathworks Inc. Natick, MA) displayed for 500 ms on a calibrated CRT monitor (ViewSonic PF817; Sony, Tokyo, Japan) via a ViSAGE (Cambridge Research Systems, Rochester, UK). Mice were positioned with their left eye perpendicular to the display, at a distance of 40 ± 1 cm. Each VEP trial was 1280 ms duration, with 100 ms before and 680 ms after stimulus presentation. Signal-averaging was performed online across 32 trials presented in each set. Four sets of 32 averaged trials were recorded at each time-point (pre- and post-stimulation) and mean voltage across the four sets used in analyses.

We used ketamine (an NMDA-receptor antagonist) as an anaesthetic during VEP recordings and LI-rTMS. Although NMDA-receptor activation is linked to offline effects of rTMS^71^, and ketamine has been shown to alter high intensity rTMS after-effects at later time points (>2 h) after stimulation^72–74^, the short time-course of experiments reported here (20 minutes post stimulation) most likely preclude downstream mechanisms of NMDA-receptor activation that involve regulation of gene expression and protein synthesis beyond immediate early genes^54,55^. Additionally, our sham-controlled experimental design avoids possible confounds due to time-varying effects of anaesthetic.

### LI-rTMS

As previously described^21^, LI-rTMS pulses were generated using a commercially available pulse generator (Global Energy Medicine, Perth Australia) modified for attachment of a custom built iron-core coil suitable for use on a mouse (8 mm outer diameter, 6 mm diameter steel bolt in the centre, 300 copper wire windings). The coil was placed in contact with the anesthetised mouse’s head, next to the V1 recording screw and near the midline, providing bilateral stimulation to the visual cortices. LI-rTMS pulses were delivered continuously for 10 minutes at 10 Hz (300 µs rise time). As described in Tang *et al*. (2016), magnetic field intensity was measured using a Hall-effect device (Honeywell 91 SS94A2D, New Jersey, USA). The peak Hall Effect voltage from the rising phase of the pulse was recorded for 4 pulses and averaged to obtain mean field strength at 1 mm intervals in perpendicular (x and y) and parallel (z) axes coordinates to a maximum of 10 mm in each plane, relative to the centre of the coil. Magnetic fields are not impeded by bone or soft tissue, making measurements in air a valid approach to estimating field strength within the brain. Hall Effect voltages were recorded and analysed with data acquisition software (Labchart 6, ADI instruments, Sydney, Australia). To confirm that the screw electrode did not interfere with intensity of the magnetic field, the Hall-effect device was positioned post-mortem within the skull directly below the coil (as described in Rodger *et al*. 2016) of a mouse with implanted screw electrodes. At V1 depth, the magnetic flux density was 10.6 mT. Since this magnetic flux density was comparable to measures at the same distance in air^21^, this indicates that the implanted screw electrode was unlikely to significantly alter the electric field induced in the brain at the level of V1. At this low magnetic field intensity (roughly 100 times less than that of rTMS used clinically), mice have no evoked motor responses or seizures.

There was no additional sensory stimulation from LI-rTMS. As previously described^21,75^, sound intensity produced by the coil (<7 dB SPL in the frequency range detectable by mice) is below the auditory threshold (30 dB SPL) to evoke electrophysiological auditory brainstem responses in both wildtype and ephrin-A2A5^−/−^ mice^76^; after 10 min stimulation, the temperature at the coil surface was 26.5 °C, and close to ambient room temperature (22–26 °C); and vibration at the coil surface could not be detected above background levels^25^. In the sham condition, all aspects of the procedure were identical but the coil was detached from the pulse-generator. This procedure ensured consistency across sham and LI-rTMS groups in time taken to place the coil, and in any minor disturbances to the mouse’s position relative to the display. Throughout all procedures the room was entirely dark and the mouse was further shielded from contaminating light from the computer screens by black curtaining on the faraday cage. A dim, indirect flashlight was used for 2–5 seconds to position the coil for LI-rTMS or sham, immediately after the pre-stimulation VEP recording, and to remove the coil immediately prior to the post-stimulation VEP recording.

### Analysis of VEP peaks

VEP peaks were designated as P1, N1, P2, N2, P3 and N3, according to standard nomenclature^28,38,77^. Analyses to define VEP peaks were semi-automated and performed blind to stimulation condition. VEP positive and negative peaks were identified using area under the curve analysis in Graphpad Prism (v.6, GraphPad Software Inc., San Diego, CA). The analysis was constrained to identify peaks greater than 10% of the maximum amplitude for each trace. The automated analysis only identified peaks which crossed baseline (0). Peaks which did not cross the baseline (i.e. the ‘shoulder’ of another peak) were defined as the point of polarity reversal and measured using Axograph (v. 1.5.4, Axon Instruments Inc., Berkeley, CA).

Consistent with previous VEP studies^38^, late VEP components showed more inter-subject variability than early components (P1, N1): all mice had a second, large, positive peak (P2), however, not all mice showed a distinct negatively deflecting peak following P2, and those without a clearly identifiable trough after P2 (‘N2’), by definition, had no clearly identifiable P3. Therefore, we quantified LI-rTMS effects on the late VEP response components, from the onset (baseline immediately preceding) of P2 to the onset of N3 in the window where P2-N2-P3 occurs. We used Axograph software to measure maximum amplitude of positively deflecting peaks, and minimum amplitude of negatively deflecting peaks and the number of distinct peaks within the P2-N2-P3 window. Traces were 100 Hz low-pass filtered, and the threshold to identify separate peaks set at >85% of adjacent peaks, and at least 10% of the maximum amplitude for each trace. This threshold level was consistent with manual identification of peaks as distinct from noise.

Negatively-deflecting peaks were identified using the same threshold criterion as described above, but using P2 as the ‘0’ baseline. After identifying distinct negatively-deflecting peaks, the measured amplitude was not corrected for P2 as baseline and thus, could be a positive or negative voltage value. Peaks were counted only if followed by a polarity reversal. In subjects where a negative-deflecting peak was present only at a single time point (pre-stimulation or post-stimulation), we analysed amplitude (µV) at the equivalent latency (ms) in the recording without a negatively-deflecting peak. This was to avoid excluding subjects showing a more extreme change in negatively-deflecting peak amplitude (e.g. disappearance or emergence of a negatively-deflecting peak). Subjects which did not have a late response negative peak at either pre- or post-stimulation were excluded in the analyses of negative peak amplitude (sample sizes shown in Fig. S1D,E). We also included average amplitude and positive peak maximum amplitude in the analysis, and confirmed that subjects with a negatively-deflecting peak were not qualitatively different from the overall cohort for other outcomes, as there were similar direction and magnitude of mean differences as in the analysis of the full cohort (data not shown).

### Immunohistochemistry

Within 5 minutes after completing recordings, mice were deeply anesthetised with 0.1 ml pentobarbitone (Lethabarb, Virbac, Australia), and transcardially perfused with 0.9% saline followed by 4% paraformaldehyde in 0.1 M phosphate buffer. The brain was dissected, post-fixed in 4% paraformaldehyde for 24 hours at 4 °C, before cryoprotection in 30% sucrose in PBS. Brains were cryosectioned coronally (40 µm) and thaw-mounted onto gelatin-subbed slides. Slides used to visualise parvalbumin-expressing cells were stored at −80 °C until processed for immunohistochemistry. Sections were brought to room temperature and allowed to air-dry for 1 h before outlining with an ImmEdge hydrophobic pen (Vector Laboratories Ltd, Peterborough, UK). Sections were permeabilised with 0.2% Triton in PBS for 20 minutes, incubated in blocking solution (10% normal donkey serum in PBS 0.2% Triton X-100) for 2 hours at room temperature and then with primary antibody (monoclonal anti-parvalbumin produced in mouse, P3088, Sigma, St. Louis, MO) diluted 1:500 in blocking solution, at 4 °C overnight. Sections were then washed in PBS (10 minutes x3) and incubated the secondary antibody (1:600 donkey anti-mouse IgG (H + L), Alexa Fluor® 488 conjugate, A-21202, Invitrogen, Carlsbad, CA) with Hoescht (1:10000) in PBS for four hours at room temperature. Sections were washed in PBS (10 minutes x2) and coverslipped with Fluoromount-G mounting medium (Southern Biotech, Birmingham, AL). Sections from different experimental groups were stained concomitantly. Adjacent sections were stained with cresyl violet and used to identify visual cortex boundaries, and to confirm electrode placement over V1, visible as a small cortical compression^28^.

Fluorescence images used for analysis of PV+ expression were taken on Nikon Eclipse 80i microscope and DS-5Mc camera (Nikon, Tokyo, Japan). Imaging and image analyses were performed blind to visual activity and stimulation conditions. Three subjects were excluded from analysis of PV+ expression due to poor staining quality (from groups: wildtype-sham visual input, and dark-delay, and one ephrin-A2A5^−/−^ with LI-rTMS with visual input). PV+ cells were counted according to stereological principles in both left and right hemispheres from 3–4 sections per subject. Region of interest boundary was defined within V1 and divided into ‘superficial’ and ‘deep’ levels, approximately corresponding to pia surface to cortical layer 4 (superficial), and layer 5 to white matter (deep). Visual cortex was identified using adjacent sections stained with cresyl violet, cross-referenced to the mouse brain atlas^69^. PV+ cells were counted only when the soma was clearly labelled, without consideration of proximal dendrite labelling or visibility. Region of interest area (mm^2^) and PV+ cell counts were measured using Photoshop CC 2015 (Adobe Systems Software, Ireland). PV+ cell density was quantified as number of PV+ cells divided by the area (mm^2^) counted from. We first confirmed that there were no significant differences between different sections or between the left and right hemispheres within the same animal (repeated measures ANOVA, p-values > 0.05, data not shown), therefore, values were averaged across sections and hemispheres to obtain mean PV+ cell density in superficial and deep layers for each subject.

### Statistics

We analysed VEP peak amplitudes using repeated measures (RM) ANOVAs with time (pre-, post-stimulation) as within-subjects factor, and visual activity type (visual input, dark), genotype (wildtype, ephrin-A2A5^−/−^) and stimulation (sham, LI-rTMS) as between-subject factors. For all RM-ANOVAs, we examined time (within-subject) by between-subject factor interactions. Significant interactions were followed up by tests for simple-interaction effects (e.g. to follow up a three-way interaction of time × genotype × stimulation tests for simple-interaction effects examined whether the time × stimulation interaction was significant in both wildtype and ephrin-A2A5^−/−^ mice). Significant simple-interaction effects were then followed up with tests for simple effects, examining group differences for one factor, within each level of every other factor (e.g. testing whether LI-rTMS and sham were significantly different at each time point, each visual input condition and each genotype). For simplicity, where multiple interactions were significant, only the highest-order interactions are reported in the text (e.g. significant 2-way interactions were not reported if qualified by a 3-way interaction). Likewise, tests for simple-interaction effects are not reported if followed-up with tests for simple-simple effects. All follow-up tests were corrected for multiple comparisons (Sidak). Number of peaks within the P2-N2-P3 window were analysed with Poisson regression. PV+ cell density measures were normally distributed despite being count data, and analysed by RM-ANOVA with cortical depth (superficial, deep) as within-subject factor and visual activity cohort, genotype and stimulation as between-subject factors. Pearson’s bivariate correlations were used to assess relationship between late response positive and negative peaks changes in amplitude values from pre- to post-stimulation. Correlations were calculated separately for each group.

Statistical analyses were performed using SPSS (v. 20, IBM Corporation, Armonk, NY). The level for significance was set at p < 0.05. The datasets analysed in the current study are available from the corresponding author on reasonable request.

## Electronic supplementary material

Supplementary Information
