# Supplementary material for: Low-intensity repetitive transcranial magnetic stimulation requires concurrent visual system activity to modulate visual evoked potentials in adult mice
Source: Sci Rep. 2018 Apr 11;8:5792. doi: 10.1038/s41598-018-23979-y (PMC5895738; doi:10.1038/s41598-018-23979-y)
Supplement: Supplementary file 1 — Supplementary Information [file 41598_2018_23979_MOESM1_ESM.pdf]

Supplementary Information

Low-intensity repetitive transcranial magnetic stimulation requires concurrent visual system activity to modulate visual evoked potentials in adult mice

Kalina Makowiecki[1,2,5], Andrew Garrett[1,2], Alan R. Harvey[1,3,4], and Jennifer Rodger[1,2,3,4]

[1]Experimental and Regenerative Neuroscience, [2]School of Biological Sciences, [3]School of Human Sciences, The University of Western Australia. [4] Perron Institute for Neurological and Translational Science, Australia. [5]Present address: Department of Systems Neuroscience, JFB, University of Goettingen, Germany.

Time in the dark and delay after stimulation

We used ANOVAs to assess separate contributions of time in the dark and delay after stimulation to interactions with genotype (wildtype, ephrin-A2A5$^{-/-}$) and stimulation condition (sham, LI-rTMS) on change in late VEP peaks (post-stimulation subtract pre-stimulation peak amplitudes). To assess the independent contribution of time in the dark we combined the two 10 min dark groups (visual input, and dark-immediate) and compared the pooled mean to the mean amplitude of the dark-delay group, which had 20 min total time in dark (i.e. treated as a single factor level in the ANOVA, Fig. S1A. To assess the contribution of delay after stimulation, we compared the dark-immediate group mean (no delay between the end of LI-rTMS and the post-stimulation VEP recording) to the mean of the combined 'delay' groups (visual input and dark-delay) in which the post-stimulation VEP was recorded 10 minutes after the end of stimulation. Significant interactions were followed up with Sidak corrected tests for simple effects, comparing the difference from pre-stimulation (post-pre) between cohorts (visual input-delay, dark-delay, and dark-immediate).

Total time in darkness between the pre- and post-stimulation VEP recordings did not significantly interact with genotype or LI-rTMS for the late response positive peak amplitudes (all interaction p-values > 0.05). The delay after stimulation did not modify how LI-rTMS affected the late response positive peak, but significantly mediated the genotype-specific amplitude changes from pre-stimulation (see Supplementary Table. 1 for interaction and main effect statistics, Fig. S1). Explaining the interaction, late response positive peak amplitudes changed from pre-stimulation differently depending on cohort in wildtypes (Fs >3.56, p-values <0.03, Fig. S1B), but not ephrin-A2A5$^{-/-}$ mice, for either stimulation condition (Fs<2.34, p-values > 0.10, Fig. S1C). Tests for simple-effects, restricted to wildtypes, showed that both the LI-rTMS+delay groups decreased positive peak amplitudes from pre-stimulation, and the magnitude of this decrease was significantly greater than the small (non-significant) decrease in the LI-rTMS dark-immediate group (vs. LI-rTMS visual input-delay, $p = 0.04$; vs. LI-rTMS dark-delay $p < 0.001$). However, the two delay groups did not have

significantly different magnitudes of decrease from pre-stimulation (LI-rTMS visual input vs. LI-rTMS dark-delay, $p = 0.24$). Furthermore, visual activity (in itself) significantly affected wildtype-sham mice: positive peak amplitudes increased from pre-stimulation with visual-input, but decreased from pre-stimulation in the dark-delay wildtype-sham mice (visual-input vs. dark-delay $p = 0.045$). The dark-immediate and visual input-delay groups were not significantly different ($p = 0.97$), and both showed a small non-significant increase from pre-stimulation.

Notably, wildtypes receiving sham during visual input had stable positive peak amplitudes between pre- and post-stimulation, but dark-stimulated wildtypes in *both* sham and LI-rTMS showed decreases in positive peak amplitudes from pre-stimulation. Considered together, this suggests that at 10 min post-stimulation, LI-rTMS decreases positive peak amplitudes to a similar extent as a period in the dark (without LI-rTMS), contrasting to the stable positive peak amplitude after visual input with sham stimulation. Thus, visual activity history (e.g. time in the dark) accounted for some of the observed changes in positive peak amplitude. However, the difference between the two dark groups (immediate vs. delay) failed to reach significance ($p = 0.11$), which therefore indicates that time in darkness is not the sole contributor to positive peak amplitude change from pre-stimulation in wildtype-sham groups.

The effect of LI-rTMS on the largest negative peak in the late response window significantly interacted with both the delay after stimulation, and time in the dark (significant 3-way and 2-way interactions, detailed in Supplementary Table 1). Follow-up interaction analyses showed that magnitude of negative peak change from pre-stimulation, the delay after stimulation and total time in the dark only significantly affected wildtypes with LI-rTMS (p-values ≤ 0.003, Fig. S1D, E). Further follow-up tests for simple effects (restricted to wildtypes with LI-rTMS) showed that the visual input-delay group had significantly greater increases in negative peak size compared to both the dark-immediate ($p = 0.01$) and dark-delay groups ($p = 0.01$), but the two dark groups did not significantly differ ($p = 0.99$).

This pattern of results is consistent with the hypothesis that LI-rTMS effects on the excitatory late response peak require visual input during stimulation, and cannot be attributed to total time in darkness or delay after stimulation.

Supplementary Table 1. Interactions (ANOVA) between delay after stimulation (immediate, delay), and total time in the dark (10min, 20min) with stimulation (LI-rTMS, sham) and genotype (wildtype, ephrin-A2A5$^{-/-}$) for late VEP response amplitude change from pre- to post-stimulation.

		Late Response			
		Positive peak (1,83)		Negative peak (1,73)	
		F	P	F	P
delay after stim. ×	genotype × stimulation	1.567	0.214	**4.268**	**0.042**
	stim.	2.854	0.095	3.667	0.059
	genotype	**8.448**	**0.005**	**4.817**	**0.031**
	[main effect]	0.548	0.461	0.000	0.984
time in dark ×	genotype × stimulation	0.136	0.714	1.148	0.287
	stim.	0.140	0.709	**4.457**	**0.038**
	genotype	0.261	0.611	**4.608**	**0.035**
	[main effect]	**13.10**	**0.001**	0.992	0.323

Statistically significant interactions shown in bold. Degrees of freedom shown in parentheses.

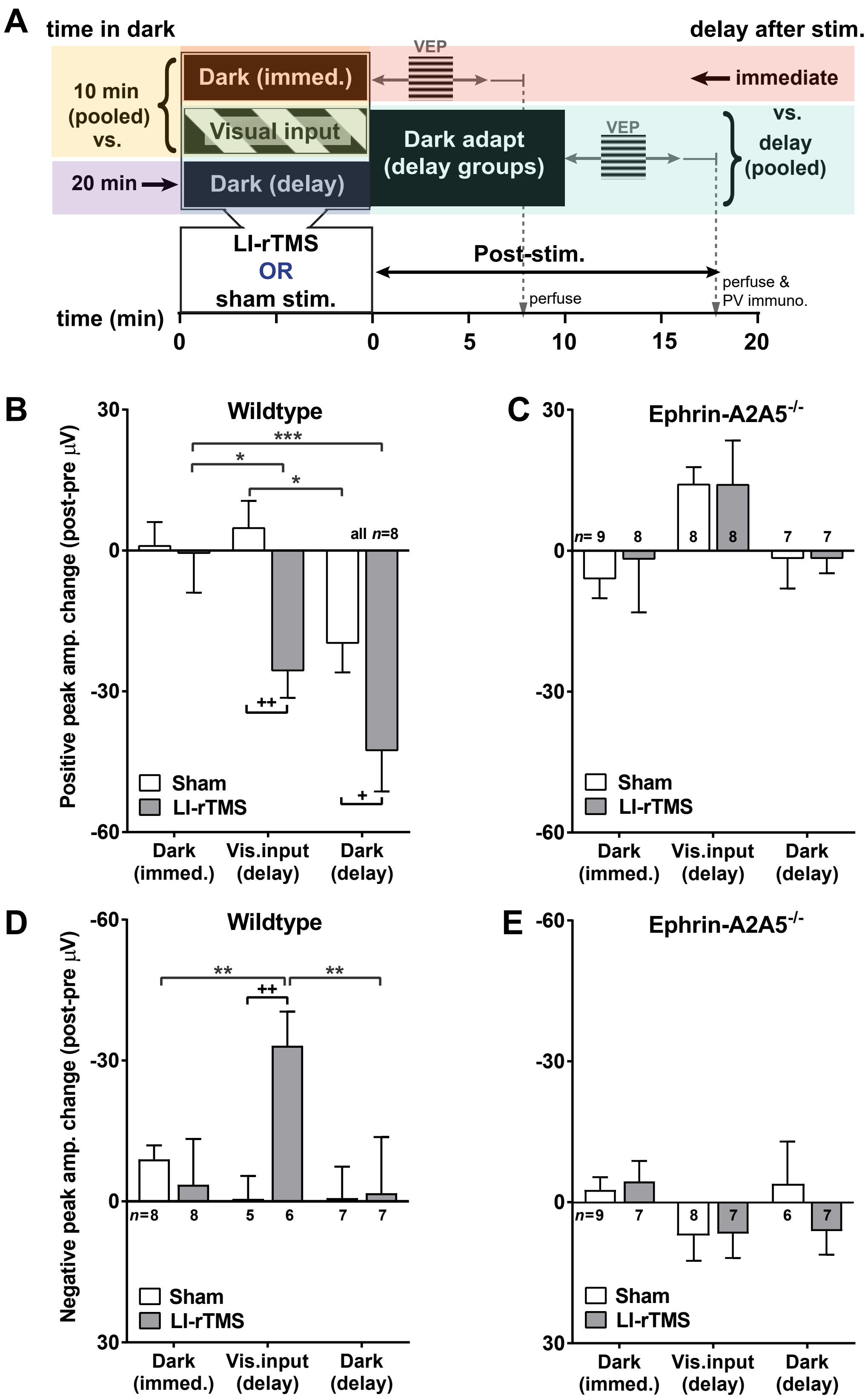

Figure S1. Contribution of time in the dark and delay after stimulation to LI-rTMS effects on late VEP response peaks. (A) Shading denotes pooling used in interaction analyses of late response VEP peaks. We assessed the independent contributions of total time in darkness between the pre-stimulation and post-stimulation VEP recordings (pooled 10min in dark vs. 20min in dark) and the contribution of delay after ending stimulation (immediate vs. pooled delay groups) on change in late VEP peaks from pre-stimulation, and interactions with genotype and LI-rTMS/sham (see Statistics section in Methods). (B-E) Mean (+SEM) change in peak amplitudes (post-pre stimulation) in largest late positive peak for wildtype (B) and ephrin-A2A5$^{-/-}$ (C), and largest late negative peak in wildtypes (D) and ephrin-A2A5$^{-/-}$ mice (E). +p<0.05 LI-rTMS vs. sham, within visual activity groups. *p<0.05, **p<0.01, ***p<0.001 comparisons between visual activity conditions.

Correlations between VEP late response peak change and PV+ cell densities in V1

We used Pearson's correlations to examine the relationship between PV+ cell densities and the change in late VEP peak amplitudes from pre to post-stimulation, separately for each group (Supplementary Table 2). Correlation analyses were run separately for PV+ cell densities in superficial (cortical layers 1-4) and deep (layers 5-6) V1, because the main analysis found that LI-rTMS with visual input significantly increased PV+ densities in superficial, but not deep, V1 layers. Although correlations are non-significant and only moderate in size, we note there was an unavoidable delay between VEP measures (recorded over a 6 minute period) and fixation of tissue for immunohistochemical staining (approximately 5 minutes after recording VEPs), limiting interpretations of correlations. Thus, it will be important for future studies to address the relationship between LI-rTMS effects on VEPs and PV+ densities using methods that allow for simultaneous measures, such as optogenetic manipulations of PV+ cells while performing electrophysiological recordings.

Supplementary Table 2. Correlations between the late VEP response amplitude change from pre- to post-stimulation and parvalbumin positive (PV+) cell densities. Pearson's r-values were calculated separately for each group, and for PV+ densities in superficial and deep V1 layers.

			Change in positive peak (r-values)			Change in negative peak (r-values)		
PV+ density			superficial	deep	n	superficial	deep	n
vis. input	wildtype	sham	-0.20	-0.10	8	0.07	0.73	5
		LI-rTMS	-0.49	-0.39	8	0.06	0.35	6
	ephrinA2A5-/-	sham	-0.49	-0.68	8	-0.16	-0.23	8
		LI-rTMS	0.50	0.31	7	0.07	-0.01	7
dark	wildtype	sham	0.43	0.29	8	-0.07	-0.15	7
		LI-rTMS	0.43	0.57	8	-0.26	-0.16	7
	ephrinA2A5-/-	sham	-0.68	-0.69	7	0.03	0.16	6
		LI-rTMS	-0.66	-0.72	7	-0.32	-0.07	7

All correlations non-significant (P >0.05, two-tailed).
